# MRI Distribution of Foot Bone Marrow Edema of Presumed Algodystrophic/BMES-Like Origin: A Small Retrospective Case Series with 3-Month Clinical Follow-Up

**DOI:** 10.3390/jcm15145736

**Published:** 2026-07-22

**Authors:** Filippo Messina, Carmelo D’Arrigo, Marco Bonifacio, Riccardo di Niccolo, Valerio Cipolloni, Marco Giuseppe Musorrofiti, Raoul Saggini, Marianna Oliva, Alberto Lo Gullo, Alessandro Conforti

**Affiliations:** 1 ASL Roma 4, 00053 Civitavecchia, Italy; filippo.messina@aslroma4.it (F.M.); carmelo.darrigo@aslroma4.it (C.D.); riccardo.diniccolo@aslroma4.it (R.d.N.); 2 Fisioterapia Medica srl, 00053 Civitavecchia, Italy; marcobonifacio1969@gmail.com; 3 University of Campania “Luigi Vanvitelli”, 81100 Caserta, Italy; valeriocipolloni@gmail.com; 4 Faculty of Health Sciences, Università Telematica eCampus, 22060 Novedrate, Italy; marcomusorrofiti@gmail.com (M.G.M.); raoulsaggini@gmail.com (R.S.); 5 Rheumatology Unit, Azienda Ospedaliera Papardo, 98158 Messina, Italy; marianna.oliva@unica.it (M.O.); albertologullo@virgilio.it (A.L.G.)

**Keywords:** bone marrow edema syndrome, foot pain, magnetic resonance imaging, algodystrophy, neridronate, retrospective cohort study

## Abstract

**Background and Objectives**: Foot bone marrow edema of presumed algodystrophic or bone marrow edema syndrome-like origin is clinically difficult to classify, and the relationship between MRI extent, pain severity, and functional impairment remains insufficiently defined. This study aimed to describe MRI distribution patterns and short-term clinical changes in a small real-world cohort of patients with persistent NSAID-resistant foot pain and presumed algodystrophic/BMES-like bone marrow edema. **Materials and Methods**: This retrospective single-center observational cohort study included 19 consecutive patients with persistent foot pain; MRI-demonstrated bone marrow edema; inadequate response to NSAIDs; management with a multimodal protocol that included neridronate, vitamin D supplementation, and, in selected patients, adjunctive physiotherapy and magnetotherapy; and available 3-month follow-up. The protocol consisted of intramuscular neridronate 25 mg once daily for 16 consecutive days, resulting in a total cumulative dose of 400 mg, together with vitamin D supplementation. Eleven patients also underwent adjunctive physiotherapy and magnetotherapy. MRI findings were categorized by anatomical location group, multilocalization, and compartment involvement. Clinical outcomes included baseline and 3-month Visual Analog Scale scores, WOMAC scores, and descriptive changes in NSAID use. Nonparametric paired and exploratory subgroup analyses were performed. **Results**: Mean age was 51.3 ± 14.9 years, and 52.6% of patients were female. MRI showed predominant midfoot involvement, with the navicular and third cuneiform most frequently affected. Median VAS changed from 8.0 at baseline to 3.0 at 3 months, and median WOMAC changed from 80.0 to 41.0; because no control group was available, these paired changes should be interpreted descriptively. Daily NSAID use decreased from 78.9% at baseline to 0% at follow-up. These findings describe clinical improvement after a multimodal management approach but do not establish the independent treatment effect of neridronate. In exploratory analyses, only two patients had multi-compartment involvement; therefore, any apparent pain-severity signal in this subgroup was considered unstable and hypothesis-generating only. Edema location group and multilocalization were not statistically associated with VAS or WOMAC outcomes. **Conclusions**: In this small retrospective single-center case series, presumed algodystrophic/BMES-like foot bone marrow edema most commonly involved the midfoot, particularly the navicular and cuneiform region. Pain scores, WOMAC scores, and daily NSAID use decreased over 3 months after multimodal care. However, the uncontrolled design, small sample size, concomitant vitamin D supplementation, adjunctive physiotherapy or magnetotherapy in many patients, and self-limiting nature of BMES-like conditions prevent attribution of these changes to neridronate or to any single treatment component. The apparent relationship between multi-compartment edema and greater pain severity was based on only two patients and should be regarded only as a weak exploratory observation.

## 1. Introduction

Bone marrow edema syndrome (BMES), also described in related literature as transient osteoporosis, transient bone marrow edema, algodystrophy, regional migratory osteoporosis, or edema-like marrow signal abnormality, is a painful but often diagnostically difficult condition of the lower extremity [1,2,3]. In the foot and ankle, the challenge is even greater because patients frequently present with persistent pain, swelling, reduced weight-bearing tolerance, and functional limitation despite relatively nonspecific clinical findings and often unremarkable or only subtly altered plain radiographs [1,2,4]. Delayed diagnosis is common, partly because the syndrome is uncommon and partly because the differential diagnosis is broad, including stress injury, osteochondral lesions, degenerative disease, infection, inflammatory arthropathy, neuropathic arthropathy, complex regional pain syndrome (CRPS), coalition, and osteonecrosis [1,5]. In real clinical practice, many patients with chronic foot pain do not fit neatly into a single strict clinical category, such as classic BMES, transient osteoporosis, or Budapest-defined CRPS, yet they still show substantial symptoms and disability. This issue is clinically relevant because foot pain itself is common, imaging-detected bone marrow edema is not rare, and the presence of edema may be associated with a slower or more prolonged painful course. In a large observational MRI series, bone marrow edema was found in 23% of patients investigated for foot and/or ankle pain, with the talus being the most frequently affected bone [6]. Although many edema patterns reflect traumatic or degenerative mechanisms, a smaller idiopathic or presumed algodystrophic subgroup remains particularly difficult to classify and manage. Prior work has also shown that edema-like abnormalities of the foot can predict long-lasting pain, with more than half of patients still symptomatic at 1 year in some imaging-defined cohorts [7]. Thus, foot bone marrow edema of presumed algodystrophic/BMES-like origin represents a clinically important problem in patients with persistent foot pain and inadequate response to NSAID therapy, especially when examination findings are inconclusive and routine classification systems do not fully capture the presentation.

**Figure 1:**
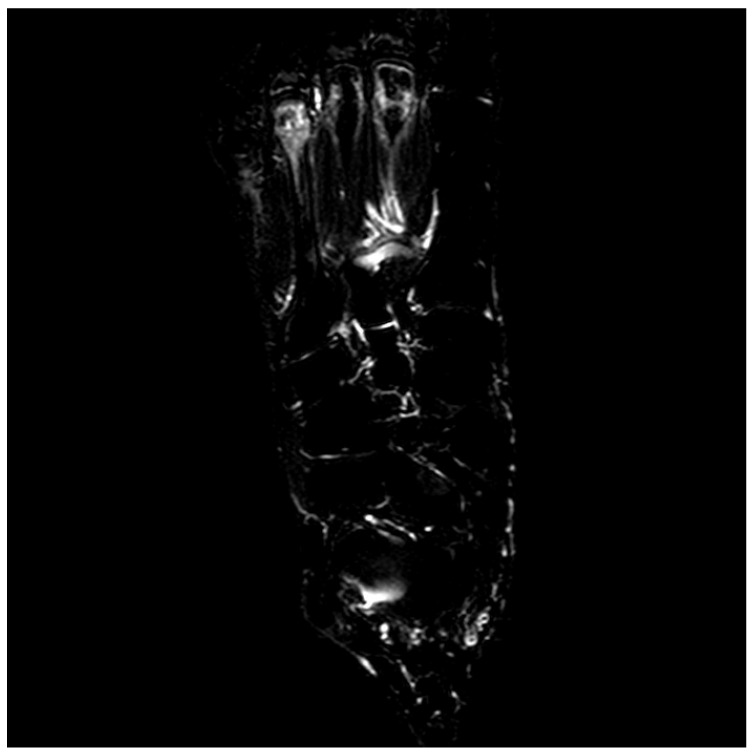
Fat-saturated T2-weighted MRI showing edema-like marrow hyperintensity at the fourth metatarsal head in a 40-year-old patient with persistent atraumatic foot pain unresponsive to NSAID therapy.

Magnetic resonance imaging (MRI) is central to the evaluation of these patients because it can detect marrow signal alteration early, map its distribution, and help distinguish diffuse, focal, subchondral, mechanical, traumatic, ischemic, or migratory patterns of disease [1,5]. The typical MRI appearance consists of low signal on T1-weighted images and high signal on fluid-sensitive or STIR/fat-suppressed T2-weighted sequences, reflecting edema-like marrow change, although this signal remains nonspecific and must always be interpreted in clinical context [1,3,6]. In the foot, MRI is particularly valuable because of the region’s complex anatomy, with multiple small bones and articulations that make localization of the pain generator difficult on examination alone [8]. Beyond simply identifying marrow edema, MRI can define whether involvement is limited to a single bone or extends across several bones, compartments, or joint regions (Figure 1). This matters because multifocal involvement is not uncommon: Singh et al. (2016) reported that more than one bone was affected in 44% of patients with foot and ankle BMES, while Orr et al. (2010) described an average involvement of three bones in their series [2,4]. Pattern analysis may also carry clinical meaning. Hirji et al. (2020) demonstrated that chronic foot pain with marrow edema can be grouped into medial, central, and lateral patterns, suggesting that edema distribution reflects underlying biomechanical or pathophysiologic stress [9]. Similarly, Cieciera et al. (2025) found that greater severity of subchondral bone marrow edema on MRI correlated with greater pain reduction after diagnostic intra-articular foot injections, indicating that MRI-defined edema burden may relate to symptom generation and treatment response [8]. At the same time, MRI helps exclude competing diagnoses rather than confirming a single syndrome in isolation. This is especially important in suspected CRPS, since Agten et al. (2020) showed that MRI could not reliably distinguish CRPS from non-CRPS foot pain, but remained useful to rule out alternative structural explanations [10]. Therefore, in patients with chronic, poorly classifiable foot pain, MRI is not only a diagnostic test but also a means of phenotyping disease extent, anatomical distribution, and potential severity.

From a therapeutic perspective, management of painful bone marrow edema has included unloading, protected weight bearing, NSAIDs, immobilization, vasoactive agents, and antiresorptive drugs such as bisphosphonates. Previous studies have suggested that pharmacologic treatment may be associated with pain reduction and MRI improvement in selected patients with painful marrow edema syndromes, although the natural course may also include spontaneous improvement over time. Neridronate, an amino-bisphosphonate, has been used in bone marrow edema and algodystrophic conditions, but evidence remains limited, especially in foot-specific real-world cohorts. Therefore, further observational data are useful, provided that treatment-related conclusions are interpreted cautiously [1,2,11,12,13,14,15,16].

Despite growing literature on marrow edema, important gaps remain. Much of the published evidence concerns the hip, knee, talus, or mixed lower-limb populations rather than foot-specific cohorts, and many reports focus on highly selected BMES cases or clearly defined etiologies rather than real-world patients with presumed algodystrophic/BMES-like edema who are difficult to classify clinically [1,13,14]. Moreover, although MRI is frequently used to document the presence of bone marrow edema, less attention has been paid to how edema distribution within the foot, such as location group, number of compartments involved, or multilocalized disease, relates to baseline pain severity and functional impairment. This is notable because prior studies suggest that edema burden and pattern may matter clinically: multi-bone disease is common [2,4], pain may persist longer in certain MRI patterns [7], edema may migrate over time [2,11,16,17], and the degree of edema on MRI may be associated with treatment-related pain relief [8]. Yet robust real-world data specifically addressing foot edema of presumed algodystrophic origin, especially in patients with chronic NSAID-resistant pain and without clean assignment to a rigid diagnostic label, remain limited. Clarifying the anatomic distribution of edema and its relationship with pain and function may improve phenotyping, aid prognostic assessment, and support more targeted treatment strategies.

This study aimed to describe the MRI distribution of foot bone marrow edema of presumed algodystrophic/BMES-like origin and to report short-term changes in pain, function, and NSAID use in a small real-world cohort managed with multimodal care. A secondary exploratory objective was to examine whether MRI-defined edema extent appeared to relate to pain severity or functional impairment. Given the retrospective, uncontrolled design, the study was intended to generate descriptive and exploratory observations rather than to test treatment efficacy or establish prognostic MRI markers.

## 2. Methodology

### 2.1. Study Design

This study was designed as a retrospective, single-center observational cohort study based on a real-world clinical dataset. The analysis focused on patients with persistent foot pain and MRI-demonstrated bone marrow edema who were managed in routine clinical practice and evaluated longitudinally after treatment. The study aimed to describe the anatomical MRI distribution of edema and to assess short-term clinical changes in pain, function, and NSAID use over a 3-month follow-up period. Accordingly, all clinical changes were interpreted as within-cohort observations and not as evidence of the independent efficacy of neridronate or any other component of care. The study was conducted in accordance with the principles of the Declaration of Helsinki and was reported in accordance with relevant STROBE recommendations for observational studies where applicable. The study was reviewed and approved by the Comitato Etico Territoriale Lazio Area 3, approval No. 7728, protocol code BIRRA, decision date 10 July 2025. Clinical and MRI data were processed in coded and de-identified form in compliance with GDPR (EU 2016/679) and Italian Legislative Decree No. 101 of 10 August 2018. No additional intervention, imaging, or patient contact was performed for study purposes.

### 2.2. Patients

Nineteen consecutive patients were included in the study. Patients were identified retrospectively from available clinical records and the institutional dataset. Inclusion criteria were: persistent foot pain; inadequate response to NSAID therapy before referral or treatment initiation; MRI evidence of bone marrow edema-like signal abnormality, defined as low signal intensity on T1-weighted images and high signal intensity on fluid-sensitive or fat-suppressed T2/STIR sequences; treatment with the standardized multimodal management approach used in the center; and availability of baseline and 3-month clinical follow-up data. Patient selection followed a consecutive retrospective case-identification approach. Records were screened from patients referred to the study center for persistent foot pain with MRI-demonstrated marrow edema during the study period. Patients were included only when all eligibility criteria were met and when baseline and 3-month clinical data were available. Patients were excluded when an alternative dominant cause of marrow edema was identified from clinical records, laboratory data, radiographs, MRI, or specialist assessment. Because the cohort was derived from a referred single-center population, selection bias and referral bias cannot be excluded.

For the purposes of this study, “presumed algodystrophic/BMES-like edema” was operationally defined as persistent symptomatic foot bone marrow edema without an alternative dominant structural, inflammatory, infectious, neoplastic, traumatic, degenerative, or osteochondral explanation in the available clinical and imaging records. Formal adjudication according to the Budapest criteria for complex regional pain syndrome was not available in the retrospective dataset. Therefore, the cohort was not classified as definite CRPS. Instead, patients were described as having presumed algodystrophic/BMES-like edema, including cases with BMES-like or CRPS-like MRI patterns but without complete prospective clinical criteria for CRPS. Patients were excluded when clinical records, laboratory findings, radiographs, MRI, or specialist assessment suggested an alternative primary etiology for marrow edema, including infection, inflammatory arthropathy, neoplastic disease, advanced degenerative joint disease, recent fracture or major trauma, osteochondral lesion, neuropathic arthropathy, or another specific diagnosis judged to better explain the MRI findings. MRI-based exclusion was based on the presence of imaging features more consistent with an alternative diagnosis, including fracture line or stress fracture pattern, osteochondral defect, advanced degenerative joint change, destructive or infiltrative marrow abnormality, features suspicious for infection or neoplasm, or a localized post-traumatic pattern judged to better explain the edema-like signal. Available records were also reviewed for relevant comorbidities and risk factors, including osteopenia or osteoporosis, overweight or obesity, diabetes, hypothyroidism, prior trauma or immobilization when documented, previous osteoporosis treatment, corticosteroid exposure when documented, inflammatory disease screening when available, and metabolic bone-related information when available.

### 2.3. Treatment Protocol

All patients were managed according to a center-based multimodal protocol that included neridronate and vitamin D supplementation, with adjunctive physiotherapy and magnetotherapy used in selected patients as part of routine care. Neridronate was administered intramuscularly at a dose of 25 mg once daily for 16 consecutive days, resulting in a total cumulative dose of 400 mg. This regimen was selected as a pragmatic outpatient protocol in the authors’ clinical setting and was based on the rationale that neridronate, an amino-bisphosphonate, has been used in painful bone marrow edema and algodystrophic conditions. Because the present study was retrospective and uncontrolled, this regimen should be interpreted as an observational evaluation of a center-based neridronate protocol rather than as proof of independent drug efficacy. All patients received vitamin D supplementation at doses exceeding 50,000 IU per month. In addition, 11 of 19 patients underwent adjunctive physiotherapy and magnetotherapy during the treatment period. Because all patients received vitamin D supplementation and many also received adjunctive physiotherapy or magnetotherapy, the observed clinical course cannot be attributed to neridronate alone or to any single treatment component.

Available records were reviewed for treatment tolerability, discontinuation, and adverse events when documented. Safety-relevant clinical information, including renal function and mineral metabolism parameters such as calcium, phosphate, and 25-hydroxyvitamin D, was extracted when available in the medical record. Because of the retrospective design, adverse-event monitoring was limited to events documented during routine care and was not based on prospective systematic surveillance.

### 2.4. Variables Collected

The following demographic, imaging, treatment, and clinical variables were extracted from the dataset: age, sex, anatomical site of edema, edema location group, edema multilocalization category, edema compartment category, comorbidities, baseline and 3-month Visual Analog Scale (VAS) scores, baseline and 3-month WOMAC scores, NSAID use at baseline and at 3 months, vitamin D supplementation, adjunctive physiotherapy or magnetotherapy, and documented treatment tolerability or adverse events when available. NSAID use was recorded descriptively from clinical documentation and patient-reported medication history in the medical record. Baseline NSAID use was categorized as daily or occasional use. At follow-up, NSAID use was categorized as daily use, occasional or reduced use, or complete discontinuation. “Occasional or reduced use” referred to patients who no longer required daily NSAIDs but still reported intermittent or lower-frequency NSAID intake during follow-up. The available dataset did not permit standardized quantification of cumulative NSAID dose, exact frequency, or use of non-NSAID analgesics across all patients.

Available records were also reviewed for relevant clinical features and safety-related information, including osteopenia or osteoporosis, overweight or obesity, diabetes, hypothyroidism, prior trauma or immobilization when documented, previous osteoporosis treatment, corticosteroid exposure when documented, inflammatory disease screening when available, renal function, calcium, phosphate, and 25-hydroxyvitamin D when available. GFI values were available only for a subset of patients and were therefore not included in the primary inferential analyses. Additional fields, such as time from diagnosis in months, history of recurrent bone marrow edema, and days lost from work, were incomplete in the available dataset and were not incorporated into the main statistical analysis.

### 2.5. MRI Classification

MRI findings were categorized according to the predefined classification system available in the dataset. The edema location group described the topographic region of involvement within the foot and was classified as forefoot, including the metatarsals and phalanges; midfoot, including the cuneiform bones, navicular, and cuboid; hindfoot, including the talus and calcaneus; or mixed distribution when more than one anatomical region was involved. The edema multilocalization category described the number of osseous locations involved and was classified as one location, two to three locations, or more than three locations. The edema compartment category described compartmental extent and was classified as involvement limited to one anatomical compartment or extending across two to three compartments.

MRI classification was based on available clinical MRI reports and retrospective review of imaging information recorded in the dataset. Because this was a retrospective real-world study, no independent prospective blinded MRI re-reading was performed for all cases, and inter-rater reliability was not assessed. Therefore, the MRI subgroup analyses should be interpreted as exploratory and dependent on the reproducibility of the classification system used in the available records. The MRI categories used in this study should therefore be regarded as pragmatic descriptive variables rather than a validated imaging classification system. They were intended to summarize anatomical distribution in routine practice and were not designed to provide quantitative edema burden, inter-reader reliability, or formal prognostic grading.

### 2.6. Outcomes

The main descriptive clinical outcomes were changes in pain and available functional status from baseline to 3 months, assessed using VAS and WOMAC scores, respectively. WOMAC was analyzed because it was available in the retrospective dataset; it was not selected as a foot-specific validated instrument for BMES-like foot disease. The available dataset for the present analysis included baseline and 3-month follow-up outcomes only. D15 and M6 outcomes were not available in this retrospective dataset and were therefore not analyzed. Secondary descriptive outcomes included the change in NSAID use between baseline and 3 months. Secondary exploratory analyses examined associations between MRI-defined edema extent and clinical outcomes. Because of missing values, GFI was not treated as a primary endpoint. Follow-up MRI was not systematically available for all patients and was therefore not analyzed as an outcome; MRI images shown in the figures are illustrative examples rather than systematically collected radiologic follow-up data.

### 2.7. Statistical Analysis

Continuous variables were summarized using sample size, minimum and maximum values, mean, standard deviation, median, and interquartile range. Categorical variables were reported as absolute frequencies and percentages. Because the cohort was small and several continuous variables did not follow a normal distribution, nonparametric methods were used for inferential testing. For consistency with these nonparametric analyses, VAS and WOMAC outcomes over time are presented primarily as median and interquartile range, whereas mean ± standard deviation is reported for baseline descriptive purposes.

Paired changes between baseline and 3-month values for VAS and WOMAC were assessed using the Wilcoxon signed-rank test, and Hodges–Lehmann median differences with 95% confidence intervals were reported. Comparisons of VAS and WOMAC across edema location groups, multilocalization categories, and compartment categories were performed using nonparametric subgroup comparisons. Ordinal associations between edema extent and clinical measures were explored with Spearman’s rank correlation coefficient and corresponding 95% confidence intervals. All subgroup and correlation analyses were considered exploratory and hypothesis-generating because of the small sample size, very small subgroup counts, and multiple comparisons. No formal adjustment for multiple testing was applied; therefore, nominal *p*-values should be interpreted cautiously and should not be considered confirmatory evidence. Analyses were conducted on a complete-case basis for each variable. No a priori sample size calculation or formal power analysis was performed because this was a retrospective analysis of all consecutive eligible patients available in the existing clinical dataset. Therefore, the study should be interpreted as descriptive and exploratory rather than confirmatory. The small cohort size and very small subgroup counts limited statistical power, precision, and the ability to distinguish statistical significance from clinical relevance. For this reason, effect estimates, confidence intervals, and the direction of observed associations were interpreted cautiously, and no definitive inferential conclusions were drawn from subgroup analyses.

## 3. Results

Nineteen patients with foot bone marrow edema of presumed algodystrophic/BMES-like origin were included in the analysis. Complete baseline and 3-month VAS and WOMAC data were available for all 19 included patients. No D15 or M6 outcome data were included in the present analysis. The results are presented in four steps: first, the baseline demographic and clinical profile of the cohort; second, the MRI distribution of bone marrow edema across individual bones and anatomical compartments; third, the short-term changes in pain, function, and NSAID use after a multimodal management approach; and fourth, exploratory associations between MRI edema extent and clinical outcomes. Descriptively, pain scores, WOMAC scores, and NSAID use decreased over 3 months. Because the study had no control group and included concomitant therapies, these changes cannot be interpreted as treatment efficacy. MRI-based subgroup analyses were exploratory only; in particular, any apparent pain-related signal for multi-compartment involvement was unstable because only two patients had multi-compartment disease.

Baseline characteristics are summarized in Table 1. The cohort was middle-aged, with a balanced sex distribution and a high baseline symptom burden. Osteopenia or osteoporosis was the most frequently recorded comorbidity group, and most patients reported daily NSAID use at study entry.

MRI distribution is summarized in Table 2. Edema most frequently involved the midfoot, particularly the navicular and cuneiform region. Most patients had localized disease, with edema confined to a single skeletal location and a single anatomical compartment.

Most patients had edema confined to a single skeletal location (57.9%), while 26.3% had involvement of two to three locations and 15.8% had more extensive involvement affecting more than three locations. Similarly, edema was limited to a single anatomical compartment in 89.5% of patients, whereas only 10.5% showed multi-compartment involvement. Overall, these findings indicate a predominance of midfoot disease with mostly localized rather than widespread MRI involvement.

**Figure 2:**
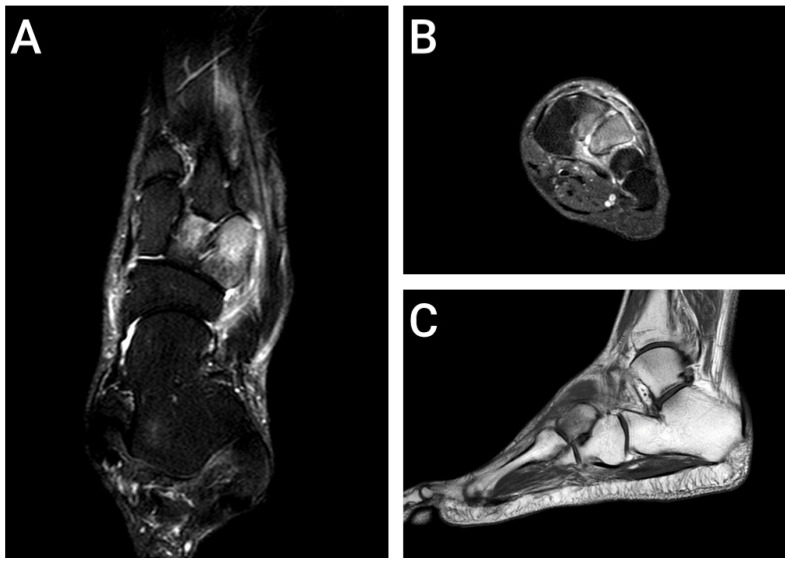
Bone marrow edema of the second and third cuneiform bones (midfoot), with mild periskeletal soft-tissue involvement, seen as hyperintensity on fat-saturated T2-weighted images in the axial (**A**) and coronal (**B**) planes and mild hypointensity on the sagittal T1-weighted image (**C**).

An illustrative midfoot MRI example is shown in Figure 2. As shown in Table 3, patients experienced substantial clinical improvement between baseline and the 3-month follow-up. Median VAS score decreased from 8.0 to 3.0, corresponding to a Hodges–Lehmann median difference of −5.0. Functional status improved in parallel, with the median WOMAC score decreasing from 80.0 at baseline to 41.0 at 3 months. Changes in NSAID use were consistent with this clinical improvement. At baseline, 78.9% of patients reported daily NSAID use, whereas no patient remained in the daily use category at follow-up. By 3 months, 57.9% reported only occasional or reduced NSAID use, and 42.1% had discontinued NSAIDs altogether. These findings describe short-term improvement after a multimodal management approach but cannot determine whether the improvement was attributable to neridronate itself, adjunctive treatment, natural disease course, or regression to the mean.

No independent prospective blinded MRI re-reading was performed for all cases, and inter-rater reliability was not assessed. The MRI classification system was simple. Table 4 summarizes the exploratory analyses of MRI edema distribution and VAS pain scores in a simplified format. No statistically significant differences were detected across edema location groups or multilocalization categories for baseline VAS, 3-month VAS, or change in VAS. The only apparent pain-related signal involved compartment involvement; however, this comparison was based on only two patients with multi-compartment edema. Therefore, this result should be read as a descriptive observation from the dataset rather than as evidence of a reproducible association.

Because only two patients had multi-compartment disease and no multiple-testing correction was applied, this association should be interpreted with substantial caution as a hypothesis-generating signal rather than confirmatory evidence. VAS improvement itself did not differ by compartment category, so the data do not support the conclusion that broader compartment involvement predicts a smaller short-term reduction in pain.

**Figure 3:**
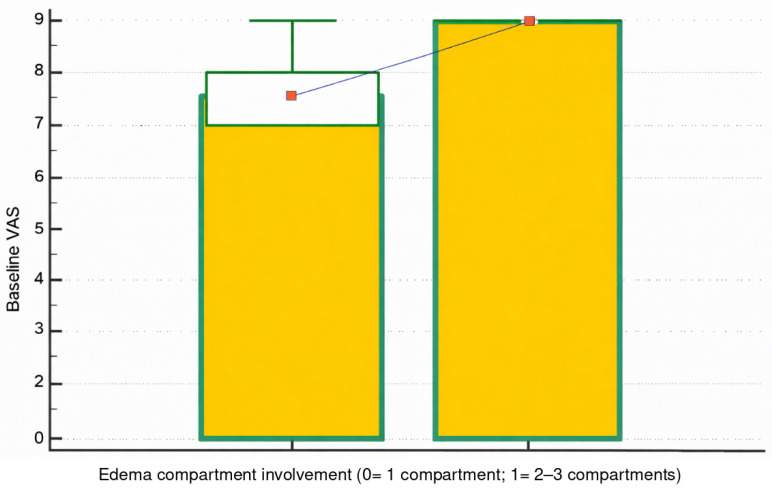
Baseline VAS according to edema compartment involvement. Multi-compartment involvement was present in only two patients; therefore, this figure is shown only to transparently display the data distribution and should not be interpreted as evidence of a reliable subgroup effect. Colors are used only to distinguish graphical elements and do not encode additional variables.

**Figure 4:**
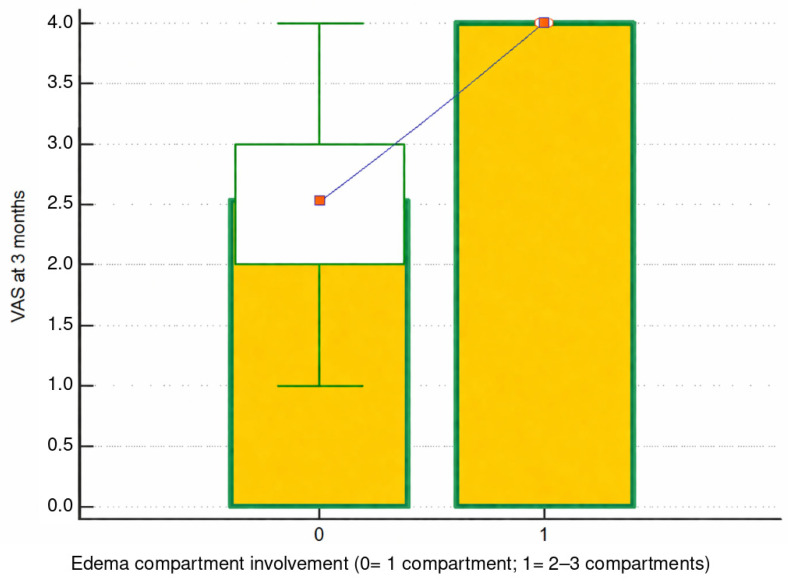
VAS at 3 months according to edema compartment involvement. Multi-compartment involvement was present in only two patients; therefore, this figure is shown only to transparently display the data distribution and should not be interpreted as evidence of a reliable subgroup effect. Colors are used only to distinguish graphical elements and do not encode additional variables.

The exploratory comparison between compartment involvement and VAS scores at baseline and follow-up is illustrated in Figure 3 and Figure 4.

Table 5 summarizes the exploratory analyses of MRI edema distribution and WOMAC functional scores in a simplified format. No statistically significant association was detected between WOMAC outcomes and edema location group, compartment involvement, or multilocalization. Given the small sample size and low statistical power, these findings should not be interpreted as evidence that MRI extent is unrelated to functional impairment; rather, no clear WOMAC-related signal was detected in the present dataset.

## 4. Discussion

This small retrospective single-center case series primarily describes MRI distribution patterns in patients with persistent NSAID-resistant foot pain and presumed algodystrophic/BMES-like bone marrow edema. The clinical follow-up data show within-cohort changes in pain, WOMAC scores, and NSAID use over 3 months after multimodal care. Because the study was uncontrolled and included concomitant vitamin D supplementation, physiotherapy, and magnetotherapy in many patients, these clinical changes cannot be attributed to neridronate or to any single intervention. Therefore, the main contribution of the study is descriptive and hypothesis-generating rather than therapeutic or prognostic.

These findings fit within, but also extend, the current literature. Most available studies have examined BMES or marrow edema in mixed foot-and-ankle samples, pediatric series, diabetic neuropathic populations, athletes with repetitive loading, or cohorts with osteoarthritis-related abnormalities rather than clinically difficult patients with chronic foot pain of presumed algodystrophic origin. The present study, therefore, adds a clinically relevant perspective by combining anatomical MRI distribution, pain and function outcomes, and exploratory severity analyses in a foot-specific cohort treated in routine practice. In that sense, the study occupies an intermediate position between descriptive MRI prevalence studies and treatment-focused series, and it helps clarify how marrow edema patterns may matter in a symptomatic, non-traumatic, and diagnostically challenging foot pain population.

### 4.1. MRI Distribution of Foot Bone Marrow Edema

**Figure 5:**
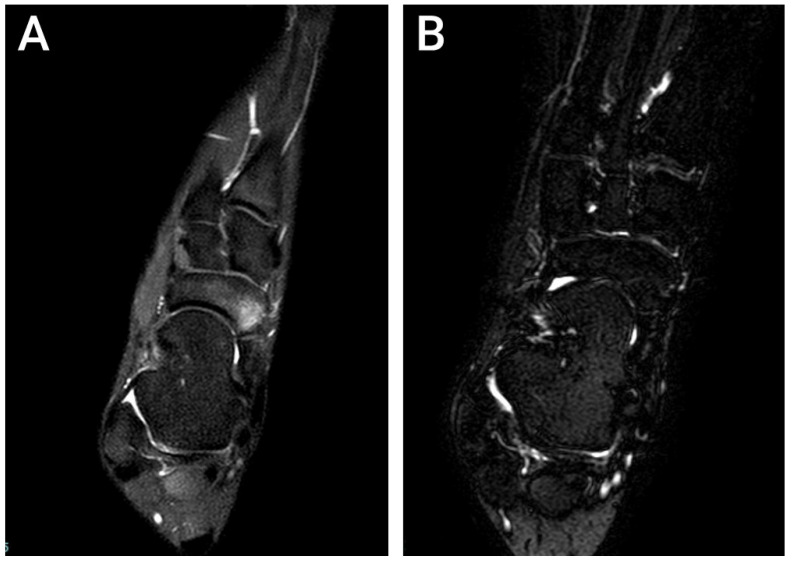
Illustrative MRI example of navicular bone marrow edema before initiation of the multimodal management approach and at 3-month follow-up. Panel (**A**) shows the baseline MRI, and panel (**B**) shows follow-up imaging in the same illustrative case. Follow-up MRI was not systematically available for all patients; this figure is shown as an illustrative example only.

The MRI distribution in our cohort was characterized by a predominance of midfoot involvement, with the navicular affected in 31.6% of patients and the III cuneiform in 26.3%, whereas the talus, although still common, was less dominant than in several previous reports (Figure 5). This contrasts with studies in which talar edema has been the leading pattern. In the series by Aigner et al. (2005), the talus was involved in 12 of 23 patients, whereas cuneiforms and metatarsals were less frequent, and navicular involvement was rare [11]. Singh et al. (2016) likewise identified the talus as the most commonly affected bone in 56% of their foot and ankle BMES cohort [2]. González-Martín et al. (2019), studying a much broader MRI population with foot and/or ankle pain, also found the talus to be the most frequently affected bone [6]. Similarly, several studies in dancers have emphasized talar dominance, especially in the talar body, neck, and posterior talus [18,19,20]. Against this background, the navicular-cuneiform predominance in our cohort may indicate that presumed algodystrophic edema in chronic foot pain represents a somewhat different clinical phenotype than the more talus-centered stress or loading-related patterns seen in dancers or the mixed etiologic populations seen in large imaging surveys.

At the same time, our findings resonate strongly with midfoot-focused MRI studies in symptomatic non-radiographic populations. Halstead et al. (2025) reported that MRI abnormalities in adults with radiograph-negative midfoot pain clustered particularly around the medial and intermediate cuneiform region, and Arnold et al. (2022) similarly found that patients with persistent midfoot pain and no radiographic osteoarthritis had bone marrow lesion patterns in the cuneiform-metatarsal joints resembling those of symptomatic midfoot osteoarthritis [21,22]. Our data, although not collected using FOAMRIS or a joint-based osteoarthritis framework, also point toward the midfoot, especially the navicular and cuneiform region, as a central anatomical substrate for clinically relevant pain. This convergence is important because it suggests that marrow edema in the midfoot may reflect a spectrum of mechanically and biologically active pathology that can be clinically meaningful even when routine radiographs or strict diagnostic labels are unhelpful.

The predominance of single-location and single-compartment involvement in our cohort also deserves comment. Most patients had edema limited to one skeletal location and one compartment, indicating that clinically important symptoms do not require diffuse whole-foot involvement. This differs from some earlier BMES series in which multifocal disease was common. Fernandez-Canton et al. (2003) found an average of 4.7 bones involved on baseline MRI, Orr et al. (2010) reported an average of three affected bones in foot and ankle BMES, and De Houwer et al. (2020) described an average of six tarsal bones involved in children with BMOS [4,23,24]. Singh et al. (2016) reported more than one bone affected in 44% of cases [2]. Our lower degree of multifocality may reflect cohort selection, classification strategy, or a different disease stage, but it also suggests that localized edema should not be dismissed as clinically trivial in chronic symptomatic patients. Importantly, the contrast between our data and these more diffuse series further reinforces the need to interpret MRI patterns within the appropriate clinical context rather than assuming that all marrow edema syndromes share the same anatomical behavior.

### 4.2. Short-Term Clinical Changes After Multimodal Care

A descriptive observation in this study was the reduction in pain scores, WOMAC scores, and daily NSAID use over 3 months. Median VAS decreased from 8 to 3, median WOMAC decreased from 80 to 41, and daily NSAID use fell from 78.9% at baseline to 0% at follow-up. These findings are consistent with previous literature suggesting that antiresorptive therapy may be associated with symptomatic improvement in painful marrow edema syndromes. Singh et al. reported faster improvement in patients treated with bisphosphonates after persistent symptoms despite initial boot treatment, while Lurati and colleagues described improvement in pain and MRI findings after intravenous neridronate in BMES cohorts [2,14,15]. Varenna et al., although studying knee osteoarthritis with bone marrow lesions rather than foot BMES, also reported pain reduction and reduced analgesic or anti-inflammatory drug use after intravenous neridronate [25].

However, the present findings must be interpreted cautiously. All patients received neridronate, but they also received vitamin D supplementation, and more than half underwent adjunctive physiotherapy and magnetotherapy. In addition, bone marrow edema syndromes and algodystrophic/BMES-like conditions may improve spontaneously or with conservative measures over time. The study also lacked an untreated or conservatively treated control group. Therefore, the observed improvement cannot distinguish the potential contribution of neridronate from adjunctive therapies, reduced loading, spontaneous recovery, natural disease fluctuation, or regression to the mean. The findings should be described as clinical changes after a multimodal management approach rather than proof of the independent therapeutic efficacy of neridronate. The present results are best interpreted as real-world observational data that justify further prospective controlled evaluation, but they should not be used to infer independent neridronate efficacy.

### 4.3. MRI Edema Extent and Pain Severity

**Figure 6:**
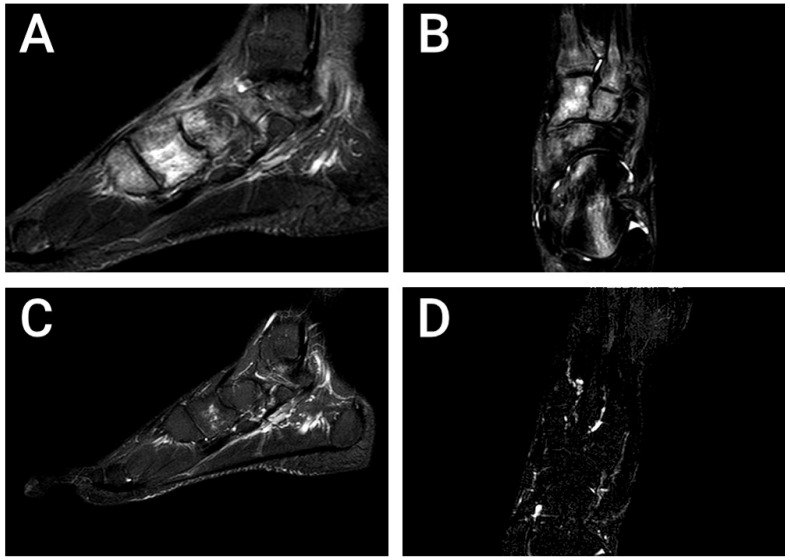
Illustrative example of multi-compartmental bone marrow edema before and after the multimodal management approach. Panels (**A**,**B**) show baseline edema distribution, while panels (**C**,**D**) show follow-up imaging in the same selected case. Follow-up MRI was not systematically available for all patients; this figure is shown as an illustrative example only.

The analysis of MRI edema extent was exploratory. In this dataset, the two patients with multi-compartment involvement had higher VAS scores than those with single-compartment disease. However, because only two patients had multi-compartment involvement and no correction for multiple testing was applied, this finding is statistically unstable and cannot support a clinical or prognostic conclusion. At most, it identifies a question for future studies: whether broader compartmental spread may relate to pain burden in larger prospectively imaged cohorts. An illustrative multi-compartment case is shown in Figure 6.

This finding is partly consistent with prior evidence that MRI-defined burden can relate to pain, but it also refines that literature by identifying a specific descriptor of burden. Elias et al. (2008) found a moderately strong correlation between talar marrow edema and pain in symptomatic ballet dancers [18]. Cieciera et al. (2022, 2025) reported that the severity of bone marrow edema on MRI correlated with immediate pain reduction after foot joint injections, implying that edema burden is linked to symptom generation [8,26]. In knee osteoarthritis, Chen et al. (2025) found that total bone marrow lesion scores correlated strongly with WOMAC pain [27]. By contrast, the present exploratory findings only raise the possibility that a simple compartment-based classification may deserve further evaluation as a descriptor of pain-related burden in chronic symptomatic foot marrow edema, because the present signal is based on only two multi-compartment cases and requires confirmation in larger cohorts before it can be considered clinically reliable. This is clinically attractive because compartment involvement is relatively easy to extract from routine MRI reporting and may be more intuitive for treatment planning than more elaborate scoring systems.

At the same time, our findings contrast with several studies showing that marrow edema is not always tightly linked to symptoms. Hatch et al. (2023) found no correlation between postmarathon marrow edema and VAS pain in long-distance runners, and all lesions resolved within 6 weeks [28]. Miskovsky et al. (2023) reported highly prevalent asymptomatic talar marrow edema in professional ballet dancers without differences in functional scores, and Katakura et al. (2024) similarly found frequent positive MRI findings in asymptomatic dancers, most of which did not become clinically important within 12 months [19,20]. Thorning et al. (2010) described remote midfoot and hindfoot marrow edema in diabetic neuropathic feet that was transient and of uncertain significance, while Chantelau et al. (2018) showed that MRI edema-equivalent changes in Charcot feet can regress, stagnate, worsen, or migrate during treatment [17,29]. Taken together, these studies caution against a simplistic interpretation of edema as a direct pain surrogate. Our data do not contradict that caution; rather, they raise the possibility that, in carefully selected symptomatic patients, the extent of involvement across compartments may carry clinical information beyond the mere presence of edema itself.

### 4.4. Pain Versus Function: Interpretation of VAS and WOMAC Findings

A notable feature of our study is the divergence between pain and functional subgroup results. While both VAS and WOMAC improved substantially at the cohort level, only VAS showed significant relationships with MRI extent variables. WOMAC was not associated with edema location group, compartment involvement, or multilocalization in the subgroup analyses, and correlation testing was uniformly non-significant. Several explanations are possible. First, VAS may simply be a more sensitive assay for detecting pain-related differences than WOMAC. Da Costa et al. (2020), in a meta-epidemiologic comparison, showed that VAS had slightly higher assay sensitivity than WOMAC pain for detecting treatment effects, without increasing between-trial heterogeneity [30]. This methodological difference may partly explain why MRI extent signals emerged more clearly for VAS than for WOMAC in our cohort.

Second, the two instruments may capture partly different constructs. Imamura et al. [31] showed in chronic knee osteoarthritis that WOMAC pain and VAS pain relate to different neural and psychological domains: WOMAC pain behaved more as an activity-related measure linked to sensory-motor and central modulation processes, whereas VAS pain behaved as a more general and psychologically influenced pain measure. Although our study concerns the foot rather than the knee, the conceptual distinction remains useful. In our cohort, MRI compartment extent may have aligned more closely with the immediate burden of perceived pain than with broader function as captured by WOMAC. A further consideration is instrument specificity. WOMAC was developed for osteoarthritis and may be less optimal for foot edema syndromes than instruments designed specifically for foot pain and disability, such as the MMFPDI or MFPDI used by Halstead et al. [21] and Arnold et al. [22]. Thus, the absence of significant WOMAC-MRI subgroup associations should not be taken to mean that function is unrelated to edema burden; rather, it may reflect a combination of sample size, construct differences, and suboptimal outcome matching. The use of WOMAC in this study also requires caution. WOMAC was originally developed and validated primarily for hip and knee osteoarthritis rather than for foot bone marrow edema or algodystrophic/BMES-like foot pain. It was used in this retrospective analysis because it was available in the clinical dataset and provided a structured measure of pain-related function. However, a foot-specific instrument would likely have been more appropriate for this population. This limitation may have reduced the sensitivity of WOMAC-based subgroup analyses and should be considered when interpreting the absence of statistically significant associations between MRI extent and functional outcomes.

### 4.5. Interpretative Considerations

The potential clinical implications of these exploratory findings are several. First, the findings are consistent with the use of MRI in chronic, difficult-to-classify foot pain not only to identify marrow edema but also to define its anatomical pattern and extent. This is consistent with imaging recommendations that regard MRI as appropriate in chronic foot pain of unclear cause, persistent post-traumatic pain with negative or equivocal radiographs, and nonradiating chronic midfoot pain of suspected osseous origin [32]. It also aligns with broader descriptive work emphasizing MRI as the modality of choice for the characterization of chronic foot and ankle pain because of its ability to depict osseous and soft-tissue pathology with high sensitivity [33]. In our cohort, MRI phenotyping was particularly useful because the patients did not fit neatly into a single rigid clinical category, yet their imaging and symptom course clearly carried therapeutic consequences. A further clinical implication is that MRI is valuable not only for detecting marrow edema, but also for differential diagnosis and patient selection. In patients with chronic foot pain and edema-like marrow signal, MRI can help exclude alternative etiologies such as inflammatory arthropathy, infection, neoplastic infiltration, degenerative disease, stress injury, or osteochondral lesions, thereby narrowing the population to those most likely to have presumed algodystrophic/BMES-like disease. In this context, MRI-based phenotyping may help describe clinically coherent subgroups for future study, but the present data do not validate MRI-based selection for any specific treatment. However, because the study was uncontrolled and included adjunctive therapies, MRI cannot be considered a validated predictive biomarker of neridronate response on the basis of these data alone.

Second, the exploratory data raise the possibility that multi-compartment edema may identify a subgroup with greater pain burden. This does not mean that more extensive edema predicts poorer treatment response, because VAS change did not differ significantly by compartment category, but it does suggest that patients with broader anatomical spread start from a worse symptomatic baseline and remain more symptomatic at follow-up. If confirmed in larger cohorts, this observation may eventually inform patient counseling; however, the present dataset is too small to support such use. It also echoes the idea from Hirji et al. (2020) that edema patterns in chronic foot pain are not random and may reflect distinct biomechanical or pathophysiologic pathways [9]. Finally, although newer techniques such as DECT virtual non-calcium imaging show promise in detecting marrow edema in active Charcot neuro-osteoarthropathy or osteoarthritis-related bone marrow lesions [27,34,35], MRI currently remains the most informative tool for the complex anatomy of the foot because it can simultaneously define lesion distribution, soft-tissue context, and differential diagnoses.

### 4.6. Strengths of the Study

This study has several strengths. It addresses an under-described real-world scenario: persistent NSAID-resistant foot pain with presumed algodystrophic/BMES-like bone marrow edema. It focuses specifically on the foot rather than on the hip, knee, or mixed lower-limb populations. It also combines anatomical MRI distribution with paired short-term clinical observations, including pain, available functional status, and NSAID use. Finally, it uses simple MRI-derived descriptors that are feasible to extract from routine reports, although these descriptors are not validated and require reproducibility testing.

### 4.7. Limitations

The limitations of this study are substantial. Selection bias is likely because the cohort was derived from a referred single-center population and included only patients with persistent symptoms, MRI-demonstrated edema, inadequate response to NSAIDs, treatment within the center-based protocol, and available 3-month follow-up. Therefore, the cohort should not be considered representative of all patients with foot bone marrow edema, acute foot pain, or conservatively managed BMES-like disease. The cohort was small, retrospective, and single-center, which limits precision, generalizability, and statistical power. In particular, only two patients had multi-compartment involvement, so the statistically significant pain-related findings for compartment extent should be interpreted as exploratory and hypothesis-generating. The study also involved multiple subgroup and correlation analyses without formal correction for multiple comparisons, increasing the possibility of false-positive findings. No a priori sample size calculation or formal power analysis was performed because the study included all consecutive eligible patients available in the retrospective dataset. This limits the ability to determine whether statistically non-significant findings reflect true absence of association or insufficient power. Similarly, statistically significant findings, particularly in very small subgroups, may not be clinically stable or reproducible. Therefore, the relationship between statistical significance and clinical relevance should be interpreted cautiously.

The lack of a control group is another major limitation. Clinical improvement after the multimodal management approach cannot be separated definitively from the natural history of marrow edema syndromes, regression to the mean, vitamin D supplementation, reduced loading, physiotherapy, magnetotherapy, or other conservative measures. Therefore, this study cannot establish the independent therapeutic efficacy of neridronate. The 3-month follow-up period is also short. It cannot establish durability of symptom change, recurrence risk, later migration of edema, or whether the observed improvement differs from the expected natural history of a self-limiting BMES-like condition.

The diagnostic classification also has limitations. Formal Budapest criteria for CRPS were not available in the retrospective dataset, and the cohort should therefore be described as having presumed algodystrophic/BMES-like edema rather than definite CRPS. Although alternative etiologies were excluded based on available clinical and imaging records, the retrospective design limited the standardization of laboratory, metabolic, inflammatory, and imaging workup across all patients. The term presumed algodystrophic/BMES-like edema was used as a pragmatic retrospective descriptor, not as a single confirmed disease entity. Although dominant alternative causes were excluded where possible, the cohort may still include biologically heterogeneous patients. This limits the clinical meaning of treating the group as a uniform diagnostic population.

MRI assessment was also limited by the retrospective nature of the study. The classification was clinically practical but not validated, and its reproducibility remains uncertain because independent blinded re-reading and inter-rater reliability testing were not performed. Follow-up MRI was not systematically available for all patients, so radiologic resolution, persistence, or migration of edema could not be analyzed. MRI examples shown in the figures should therefore be interpreted as illustrative cases rather than systematic imaging outcomes. In addition, the MRI classification system used in this study was not externally validated, and its interobserver reproducibility remains unknown.

Outcome measurement also has limitations. WOMAC was available in the dataset and provided a structured functional measure, but it was originally developed for hip and knee osteoarthritis and may be less sensitive than foot-specific disability instruments for this condition. NSAID use was recorded descriptively from clinical records and patient-reported medication history, but exact dose, frequency, cumulative exposure, and non-NSAID analgesic use were not consistently available. Finally, adverse-event assessment was limited to documentation in routine care records and was not based on prospective systematic safety monitoring.

### 4.8. Future Directions

Future studies should build on these findings in several ways. Prospective multicenter cohorts with larger numbers would allow more stable subgroup comparisons and better assessment of whether compartment-based MRI phenotyping truly predicts symptomatic burden or longer-term outcomes. Serial MRI could clarify whether the patients with higher pain and multi-compartment involvement also show slower radiologic regression, more frequent migration, or recurrence, as reported in prior BMES and Charcot literature [17,23,24]. Comparative studies of neridronate against standardized conservative treatment would help determine how much of the observed improvement is attributable to drug effect rather than spontaneous resolution. It would also be valuable to incorporate foot-specific outcome measures and structured MRI scoring systems to better align imaging severity with pain and disability. In summary, the present findings suggest that MRI-defined compartmental spread may be clinically relevant in chronic painful foot marrow edema, but this observation remains exploratory because of the very small number of multi-compartment cases. Similarly, the observed short-term clinical improvement after a multimodal management protocol requires confirmation in prospective controlled studies before conclusions can be drawn about the independent efficacy of neridronate.

## 5. Conclusions

This small retrospective single-center case series describes MRI distribution patterns and short-term clinical changes in patients with chronic NSAID-resistant foot pain and presumed algodystrophic/BMES-like bone marrow edema. Edema most commonly involved the midfoot, particularly the navicular and cuneiform region. Pain scores, WOMAC scores, and daily NSAID use decreased over 3 months after multimodal care that included neridronate, vitamin D supplementation, and adjunctive physiotherapy or magnetotherapy in many patients. Because the study was uncontrolled, retrospective, small, and potentially affected by selection bias, natural history, regression to the mean, and concomitant therapies, these changes cannot be attributed to neridronate or to any single treatment component. The apparent association between multi-compartment edema and greater pain severity was based on only two patients and should be regarded as a weak exploratory observation rather than a prognostic finding. Larger prospective controlled studies using standardized diagnostic criteria, blinded MRI review, serial imaging, and validated foot-specific outcome measures are required before conclusions can be drawn regarding treatment efficacy, MRI prognostic value, or generalizability.

## Figures and Tables

**Table 1:** Baseline demographic and clinical characteristics of the study cohort (*n* = 19).

Variable		Overall Cohort
Age (years)	mean ± SD	51.3 ± 14.9
	median (IQR)	56.0 (47.5–61.5)
Sex	Male	9 (47.4%)
	Female	10 (52.6%)
Comorbidities		
Osteopenia/osteoporosis		7 (36.8%)
Overweight/obesity		5 (26.3%)
Diabetes		1 (5.3%)
Hypothyroidism		1 (5.3%)
Other or no recorded comorbidity		5 (26.3%)
Baseline VAS	mean ± SD	7.71 ± 0.77
	median (IQR)	8.0 (7.0–8.0)
Baseline WOMAC	mean ± SD	80.42 ± 3.22
	median (IQR)	80.0 (78.0–82.75)
Baseline NSAID use		
Daily use		15 (78.9%)
Occasional use		4 (21.1%)

Abbreviations: IQR, interquartile range; NSAID, nonsteroidal anti-inflammatory drug; VAS, Visual Analog Scale; WOMAC, Western Ontario and McMaster Universities Osteoarthritis Index. Note: Continuous variables are presented as mean ± standard deviation and median (IQR); categorical variables are presented as *n* (%). “Other or no recorded comorbidity” includes patients with chronic plantar fasciitis, hyperuricemia, or no comorbidity recorded in the dataset.

**Table 2:** Anatomical distribution and extent of foot bone marrow edema on MRI.

Individual Bone Involvement		
MRI variable	*n*	%
I metatarsal	3	15.8
II metatarsal	3	15.8
III metatarsal	3	15.8
IV metatarsal	2	10.5
V metatarsal	2	10.5
Phalanges	1	5.3
I cuneiform	3	15.8
II cuneiform	3	15.8
III cuneiform	5	26.3
Cuboid	1	5.3
Navicular	6	31.6
Talus	4	21.1
Calcaneus	2	10.5
MRI distribution categories		
Forefoot	3	15.8
Midfoot	10	52.6
Hindfoot	4	21.1
Multilocalization		
1 location	11	57.9
2–3 locations	5	26.3
>3 locations	3	15.8
Compartment involvement		
1 compartment	17	89.5
2–3 compartments	2	10.5

Note: Percentages are calculated using the full cohort denominator (*n* = 19). For individual bone involvement, percentages do not sum to 100% because some patients had edema involving multiple bones. Multi-compartment involvement is reported under “Compartment involvement.”

**Table 3:** Changes in pain, function, and NSAID use from baseline to 3 months.

Outcome	Baseline	3 Months	Effect Estimate/Median Difference	95% CI	*p* Value
VAS score, median (IQR)	8.0 (7.0–8.0)	3.0 (2.0–3.0)	−5.0	−5.0 to −5.0	<0.0001
WOMAC score, median (IQR)	80.0 (78.0–82.75)	41.0 (38.0–43.75)	−39.5	−40.0 to −39.0	<0.0001
NSAID use, *n* (%)					
Daily use	15 (78.9%)	0 (0.0%)	-	-	-
Occasional or reduced use	4 (21.1%)	11 (57.9%)	-	-	-
Complete discontinuation	0 (0.0%)	8 (42.1%)	-	-	-

Abbreviations: CI, confidence interval; IQR, interquartile range; NSAID, nonsteroidal anti-inflammatory drug; VAS, Visual Analog Scale; WOMAC, Western Ontario and McMaster Universities Osteoarthritis Index. Note: Effect estimates for VAS and WOMAC are Hodges–Lehmann median differences from paired Wilcoxon signed-rank tests. VAS and WOMAC are presented as median and interquartile range because paired comparisons were performed using nonparametric methods. NSAID use is presented descriptively only. Baseline NSAID use was categorized as daily or occasional use from clinical records and patient-reported medication history. At follow-up, “occasional or reduced use” indicated that the patient no longer required daily NSAIDs but still reported intermittent or lower-frequency NSAID intake. Exact NSAID dose, cumulative exposure, and non-NSAID analgesic use were not consistently available.

**Table 4:** Summary of exploratory associations between VAS scores and MRI edema distribution.

MRI Analysis	Baseline VAS	3-Month VAS	VAS Change	Interpretation
Location group	Ht = 5.6849; *p* = 0.128	Ht = 4.8377; *p* = 0.184	Ht = 0.3346; *p* = 0.953	No significant difference by location group.
Compartment involvement	Ht = 5.2499; *p* = 0.0219	Ht = 4.4772; *p* = 0.0344	Ht = 0.0437; *p* = 0.834	Numerically higher VAS in only two multi-compartment cases; very unstable exploratory observation, not suitable for clinical inference.
Multilocalization	Ht = 5.1615; *p* = 0.0757	Ht = 4.4118; *p* = 0.110	Ht = 0.2704; *p* = 0.874	No significant association by multilocalization category.
Compartment correlation	rho = 0.54; *p* = 0.0170	rho = 0.499; *p* = 0.0297	rho = −0.0493; *p* = 0.8412	Exploratory pain-severity signal only; not confirmatory.
Multilocalization correlation	rho = 0.303; *p* = 0.2074	rho = 0.298; *p* = 0.2147	rho = −0.118; *p* = 0.6293	No significant correlation with VAS outcomes.

Abbreviations: Ht, Kruskal–Wallis statistic; VAS, Visual Analog Scale. Note: The table was simplified to improve readability in this small exploratory cohort. Detailed subgroup medians were removed from the main table; all subgroup and correlation analyses remain hypothesis-generating because subgroup counts were very small and *p* values were unadjusted. Because only two patients were in the multi-compartment category, the nominal *p* values for this comparison should not be interpreted as evidence of a reliable association.

**Table 5:** Summary of exploratory associations between WOMAC scores and MRI edema distribution.

MRI Analysis	Baseline WOMAC	3-Month WOMAC	WOMAC Change	Interpretation
Location group	Ht = 5.4054; *p* = 0.1444	Ht = 4.5052; *p* = 0.2118	Ht = 1.9239; *p* = 0.5884	No significant difference by location group.
Compartment involvement	Ht = 0.8700; *p* = 0.3509	Ht = 0.8700; *p* = 0.3509	Ht = 1.7751; *p* = 0.1828	No significant association by compartment involvement.
Multilocalization	Ht = 0.1556; *p* = 0.9252	Ht = 0.3809; *p* = 0.8266	Ht = 3.1398; *p* = 0.2081	No significant association by multilocalization category.
Compartment correlation	rho = −0.220; *p* = 0.3658	rho = −0.220; *p* = 0.3658	rho = 0.314; *p* = 0.1904	No significant correlation with WOMAC outcomes.
Multilocalization correlation	rho = −0.0596; *p* = 0.8087	rho = −0.107; *p* = 0.6623	rho = 0.375; *p* = 0.1138	No significant correlation with WOMAC outcomes.

Abbreviations: Ht, Kruskal–Wallis statistic; WOMAC, Western Ontario and McMaster Universities Osteoarthritis Index. Note: The table was simplified to improve readability in this small exploratory cohort. Results should not be interpreted as confirmatory because subgroup counts were small, analyses were unadjusted, and WOMAC may be less sensitive than foot-specific outcome measures for this population.

## Data Availability

The data presented in this study are available on request from the corresponding author. The data are not publicly available due to patient privacy and institutional and ethical restrictions.
